# A Highly Sensitive ELISA and Immunochromatographic Strip for the Detection of *Salmonella typhimurium* in Milk Samples

**DOI:** 10.3390/s150305281

**Published:** 2015-03-04

**Authors:** Wenbin Wang, Liqiang Liu, Shanshan Song, Lijuan Tang, Hua Kuang, Chuanlai Xu

**Affiliations:** State Key Lab of Food Science and Technology, School of Food Science and Technology, Jiangnan University, Wuxi 214122, China; E-Mails: wenbin66@yeah.net (W.W.); raxray@gmail.com (L.L.); songshanshan0626@126.com (S.S.); tlj844944327@163.com (L.T.); kuangh@jiangnan.edu.cn (H.K.)

**Keywords:** *Salmonella typhimurium*, flagellin, LPS, monoclonal antibody, sandwich ELISA, immunochromatographic strip, detection

## Abstract

Murine monoclonal antibodies to target *Salmonella typhimurium* flagellin and lipopolysaccharide (LPS) were prepared and characterized. For the immunological detection of *S. typhimurium*, different pairs of monoclonal antibodies (MAbs) were tested in a sandwich enzyme linked immunosorbent assay (ELISA) format. After comparison, a sandwich ELISA and immunochromatographic strip based on LPS MAbs was established to detect *S. typhimurium*. The determination limits of the immunochromatographic strip in phosphate-buffered saline (PBS) containing 0.1% Tween 20 (PBST) and pure milk sample were found to be 1.25 × 10^5^ colony-forming units (cfu)/mL and 1.25 × 10^6^ cfu/mL *S. typhimurium*, respectively. Results can be obtained with the naked eye in 10 min. Cross-reactivity was observed with *Salmonella paratyphi* B, but not *S. paratyphi* A or *Salmonella enteritidis*. The LPS MAbs based immunochromatographic strip is rapid and convenient to detect *S. typhimurium* in milk samples.

## 1. Introduction

Salmonella, a globally distributed foodborne pathogen, is a leading cause of human gastroenteritis [1]. The presence of salmonella originating from egg, chickens, and dairy products has been frequently reported [1,2]. Based on the O and H antigens, salmonella can be divided into more than 2500 serotypes [3]. Among of these serotypes, the subspecies *enterica*, especially *Salmonella enterica* serovarTyphimurium and *Salmonella enterica* serotypeEnteritidis, has been most often linked to human salmonellosis cases [4,5].

The gold standard detection method for salmonella is culturing, which is laborious and time consuming. Rapid detection methods, such as polymerase chain reaction (PCR) and immunoassay methods, have been widely reported [6,7]. PCR, although very rapid and sensitive, suffers from the effects of inhibitory substances, high cost, and the need for professionally trained operators [6,8]. Alternatively, immunoassay methods such as ELISA and immunochromatographic strip were considered to be a powerful tool for the detection of toxins and food borne pathogens [9,10,11].

Antibodies are the cornerstone of and immunoassay method and dictate its sensitivity and specificity. Salmonella main surface antigens, such as lipopolysaccharide (LPS, O antigen) and flagellin (H antigen), have been studied and monoclonal antibodies (MAbs) have been generated [12,13,14]. However, most of the previous works studied the specificity of the MAbs and ELISA methods to detect salmonella were seldom developed. Sadallah and coworkers developed a sandwich ELISA for *S. typhi* with a rabbit polyclonal antibody as a capture antibody and a flagellin mAb as a detection antibody [15]. The sensitivity of this sandwich ELISA for flagellin and salmonella cells is 5–10 ng/mL and 10^4^–10^5^ cfu/mL, respectively. Based on a genus-specific LPS mAb T6, Tsang and colleagues developed a sandwich ELISA which can detect 1 ng/ml Ra LPS (having a complete core oligosaccharide without O-specific chains) and 10^6^ cfu/mL *S. typhimurium* [16].

Although flagellin and LPS MAbs against salmonella have been produced, it’s still not known which kind of antigen is more suitable for detection of *S. typhimurium* in a sandwich ELISA. Herein, we generated MAbs to *S. typhimurium* flagellin and LPS. These MAbs were paired in a sandwich ELISA format and different mAb pair combinations were compared. Based on this, a highly sensitive ELISA and immunochromatographic strip were developed to detect *S. typhimurium* in spiked pure milk.

## 2. Materials and Methods

### 2.1. Strains and Growth Conditions

*Salmonella enterica* serovarTyphimurium (*S. typhimurium*, ATCC 13311), *Escherichia coli* O157, *Staphylococcus aureus*, *Listeria monocytogenes*, and *Cronobacter sakazakii* (ATCC 29544) were obtained from the Center of Industrial Culture Collection (CICC, Beijing, China)*. Salmonella enterica* serovar Paratyphi B (*S. paratyphi* B, CMCC 50094) was obtained from the National Center for Medical Culture Collections (CMCC, Beijing, China). *Salmonella enterica* serotype Enteritidis (*S. enteritidis*, ATCC 13076), *S. typhimurium* (ATCC 14028), and *Campylobacter jejuni* (ATCC 49443) were kindly provided by the Hunan Entry-Exit Inspection and Quarantine Bureau (Changsha, China). All bacteria were cultured overnight in Brain-Heart Infusion (BHI) broth at 37 °C and concentrations were obtained by traditional plate counting method.

### 2.2. Purification and Characterization of S. typhimurium Flagellin

*S. typhimurium* flagellin was purified as described by Ibrahim and colleagues [12]. Protein concentrations were determined using the Bradford assay. The extract was characterized by sodium dodecyl sulfate-polyacrylamide gel electrophoresis (SDS-PAGE) with a stacking gel and separating gel containing 5% and 10% acrylamide, respectively. Furthermore, Salmonella H:i standard sera (Statens Serum Institute, Copenhagen, Denmark) was used to characterize the purified flagellin by indirect ELISA. The indirect ELISA was conducted as previously described [17].

### 2.3. Monoclonal Antibodies for Detecting S. typhimurium Flagellin and LPS

For immunizations, 6- to 8-week-old BALB/c mice were subcutaneously injected with the prepared antigen (emulsified in Freund’s adjuvant). The dose for the three immunizations of flagellin was 80, 80 and 40 μg, respectively. Smooth-type LPS from *S. typhimurium* (Sigma, Saint Louis, MO, USA) was mixed with *S. typhimurium* cells (boiled for 10 min) and the dose for each immunization was 100 μg LPS with 10^8^ *S. typhimurium* cells, 100 μg with 10^8^ cells, and 50 μg LPS with 5 × 10^7^ cells; 7 days after the third immunization, the mouse with the highest titer was sacrificed for cell fusion. Positive cells were selected against purified flagellin or LPS by indirect ELISA and were subcloned by limiting dilution. Ra LPS from *S. typhimurium* SL1181 (Sigma) was used to study the cross-reactivity of the selected MAbs with indirect ELISA. The isotype of each antibody was determined using an Antibody Isotyping Kit (Envirologix, Portland, ME, USA). HRP-conjugated antibodies were prepared as previously described [17].

### 2.4. The Use of a Combination of Flagellin and LPS MAbs to Develop a Sandwich ELISA for Detecting S. typhimurium

For immunodetection of *S. typhimurium*, all flagellin and LPS MAbs were paired in a sandwich format. Using one *S. typhimurium* mAb to coat plates as a capture antibody and another HRP-conjugated *S. typhimurium* mAb as a detection antibody, *S. typhimurium* cells could be detected by the most suitable pair. We studied four types of pairwise reactions with flagellin and LPS MAbs. Selected pairs with a high positive/negative (P/N) value were selected and compared pairs in terms of sensitivity for the detection of *S. typhimurium*. Sandwich ELISA was performed as previously described [10,18]. 

### 2.5. The Establishment and Cross-Reactivity of LPS mAb-Based Sandwich ELISA

The selected mAb pair was optimized for the concentration of coating antibody, detection antibody, and Tween 20 in dilution buffer. A standard curve for *S. typhimurium* was established after optimization. The sandwich ELISA that we established was then assessed by specificity studies and with artificially spiked milk samples. For specificity studies, pure cultured *E. coli*, *S. aureus*, *L. monocytogenes*, *C. jejuni*, *S. typhimurium*, *S. paratyphi* B, *S. paratyphi* A, *S. enteritidis*, and *Cronobacter sakazakii* were tested at 10^8^ cfu/mL. 

### 2.6. LPS mAb-Based Immunochromatographic Strip for S. typhimurium

The immunochromatographic strip detects *S. typhimurium* in a sandwich format. L2 mAb and goat anti-mouse IgG were immobilized on a nitrocellulose (NC) membrane (1 μL/cm) with a dispenser as test line and control line, respectively. L6 mAb was labeled with gold nanoparticles and stored in 0.02 M PBST at 4 °C until use. The colloidal gold (25 nm in diameter) and gold-antibody conjugate were prepared as previously described [19]. The concentration of L2 mAb used for the test line, concentration of L6 mAb to conjugate gold nanoparticles and the reaction time were optimized. For detection, 150 μL standard solutions or samples was load in the reaction buffer containing 7 μL gold-labeled L6 mAb and 43 μL PBST. Then the sample pad of the strip was immersed in the reaction buffer and allowed to react. Result was visualized with naked eye. 

### 2.7. Milk Samples

Pure milk from the local supermarket was artificially spiked with freshly cultured *S. typhimurium* at four different levels (the concentrations were unknown). The spiked samples were then simultaneously detected by ELISA, immunochromatographic strip and a traditional plate counting method. Plate counting was performed as previously described [20]. We loaded 100 μL of the spiked milk samples on the ELISA plate without any dilution or centrifugation. For a standard curve of sandwich ELISA, previously cultured *S. typhimurium* (3 × 10^9^ cfu/mL) was used as a standard and diluted in pure milk, resulting in a series of concentrations as follows: 6 × 10^7^, 2 × 10^7^, 6.67 × 10^6^, 2.22 × 10^6^, 7.41 × 10^5^, 2.47 × 10^5^, 8.23 × 10^4^, 2.74 × 10^4^ and 9.14 × 10^3^ cfu/mL. We loaded 100 μL of each standard in milk solution on the plate. Pure milk without spiked *S. typhimurium* was used as a control. The concentration of *S. typhimurium* in the spiked’ milk sample was calculated based on the standard curve. The determination limit means the corresponding concentration of the standard when the absorbance was 2.1 times the mean value of the blank values. For the immunochromatographic strip, we loaded 150 μL spiked milk samples into the reaction buffer containing 7 μL gold-labeled L6 mAb and 43 μL PBST.

## 3. Results and Discussion

### 3.1. Characterization of the S. typhimurium Flagellin Preparation

**Figure 1:**
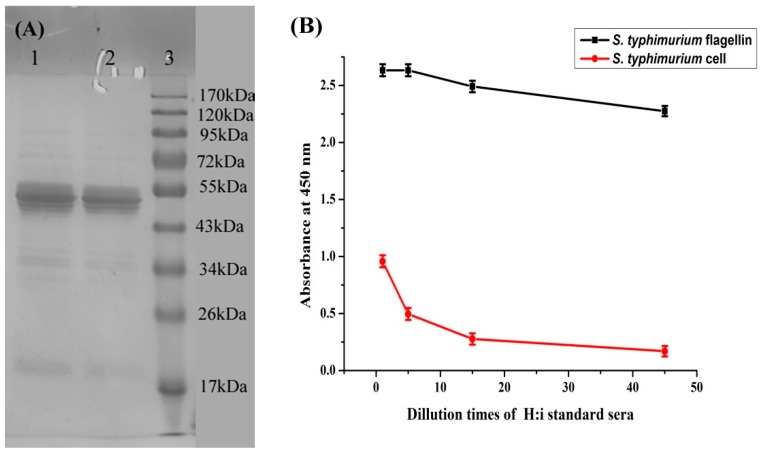
(**A**) SDS-PAGE of prepared flagellin from *S. typhimurium*. Line 1 and 2: Flagellin sample. Line 3: Standard weight protein Maker; (**B**) Characterization of *S. typhimurium* flagellin preparation by indirect ELISA. Red line is control with *S. typhimurium* cell as coating antigen.

Purified *S. typhimurium* flagellin was characterized by SDS-PAGE and indirect ELISA. Figure 1A shows that the flagellin preparation yielded a major band between 43 and 55 kDa. This result is comparable with Ibrahim’s finding that salmonella flagellin preparations had a molecular weight that ranged from 47.7 to 58.4 kDa [12].

Figure 1B shows that SSI standard H:i sera strongly reacted with the *S. typhimurium* flagellin preparation, and also reacted to *S. typhimurium* cells, although at a low level. This finding indicates that the purified flagellin is a phase 1 flagellar antigen (H:i).

### 3.2. Characterization of S. typhimurium Flagellin and LPS MAbs

After cell selection, we obtained four *S. typhimurium* flagellin MAbs, named F1, F2, F3 and F4, and six *S. typhimurium* LPS MAbs, named L1, L2, L3, L4, L5 and L6. The cross-reactivity of the selected *S. typhimurium* MAbs with viable *S. typhimurium* and *S. enteritidis* was tested by indirect ELISA. *S. typhimurium* LPS MAbs were tested for cross-reactivity with Ra LPS from *S. typhimurium* SL1181. The isotype of each antibody was determined using an isotyping kit (results are shown in Table 1).

**Table 1:** Isotype and cross-activity of *S. typhimurium* flagellin MAbs and LPS MAbs.

	F1 IgG1	F2 IgG1	F3 IgG1	F4 IgG1	L1 IgG2b	L2 IgG2b	L3 IgG1	L4 IgG2b	L5 IgM	L6 IgG1
***S. typhimurium* ATCC 13311**	++	+	+	+	+++	+++	+++	+++	+++	+++
***S. typhimurium* ATCC 14028**	+	+	+	-	+++	+++	+++	+++	+++	+++
***S. paratphi* B CMCC 50094**	-	-	-	-	+++	+++	+++	+++	+++	+++
***S. enteritidis* ATCC 13076**	-	-	-	-	-	-	-	-	-	-
**LPS**					+++	+++	+++	+++	+++	+++
**Ra LPS**					-	-	-	-	-	-

Table 1 shows that *S. typhimurium* flagellin MAbs react with two *S. typhimurium* strains (Phase 1: i and Phase 2: 1, 2) and do not cross react with *S. paratyphi* B (Phase 1: b and Phase 2: 1, 2) or *S. enteritidis* (Phase 1: g, m, Phase 2: -). This might be because the salmonella flagellin tends to elicit specific antibodies in animals [21], although common antigenic determinants do exist, as reported by Van Asten and coworkers [22]. However, all selected *S. typhimurium* LPS MAbs react with two *S. typhimurium* strains (O antigen 1,4,12) and cross react with *S. paratyphi* B (O antigen 1,4,12), but not *S. enteritidis* (O antigen 1,9,12). Furthermore, all of these LPS MAbs do not react with Ra LPS. These findings indicate that all of the selected *S. typhimurium* MAbs may recognize the O-specific chain of *S. typhimurium*. Therefore, LPS mixed with salmonella cells or LPS-coated salmonella cells might tend to produce specific antibodies to the O-specific chain in mice [23]. 

### 3.3. A Combination of Flagellin and LPS MAbs for the Detection of S. typhimurium

We studied four pairwise mAb combinations. Table 2 shows that when *S. typhimurium* flagellin MAbs are used as both the capture and detection antibodies, only F1-F1HRP and F3-F1HRP could be paired. When *S. typhimurium* flagellin MAbs were used as a capture antibody and *S. typhimurium* LPS MAbs are used as a detection antibody, all combinations tested, except for two, could be paired. When *S. typhimurium* LPS MAbs were used as both capture and detection antibodies, all combinations except for one could be paired. However, when *S. typhimurium* LPS MAbs were used as a capture antibody and *S. typhimurium* flagellin MAbs were used as a detection antibody, no combinations tested could be paired. Because these MAbs tend to be specific for *S. typhimurium*, the specificity was not studied between different mAb pairs. Different types of pairs were further compared for their sensitivity. F1-F1HRP, F1-L6HRP and L2-L6HRP represent the three most specific pairs that we tested.

**Table 2:** Pairwise study of *S. typhimurium* flagellin MAbs and LPS MAbs in sandwich format. Viable *S. typhimurium* was used as standard with a concentration at 10^7^ cfu/mL. P/N value below 2.1 was not shown.

	F1	F2	F3	F4	L1	L2	L3	L4	L5	L6
**F1-HRP**	7.68		7.24	4.93						
**F2-HRP**										
**F3-HRP**			2.28							
**F4-HRP**										
**L1-HRP**	7.72	6.37	6.93	6.42	9.62	14.18	13.88	10.03	11.55	12.45
**L2-HRP**	14.88	9.75	19.19	14.80	15.42	14.80	17.47	17.38	10.95	15.47
**L3-HRP**	2.34		2.42		3.30		9.38	2.86	10.48	8.30
**L4-HRP**	10.12	7.16	10.57	7.91	14.81	14.50	16.66	15.10	17.37	15.65
**L5-HRP**	16.41	12.81	14.15	12.3	20.19	17.14	19.51	20.38	17.27	12.58
**L6-HRP**	18.35	7.81	13.83	6.47	17.63	22.66	22.37	21.00	20.50	20.09

**Figure 2:**
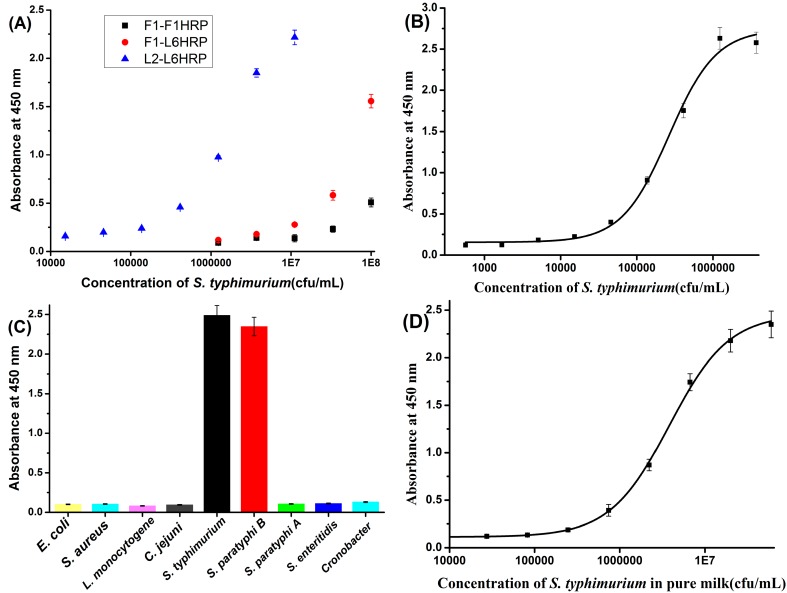
(**A**) Sensitivity of the three types of pair in sandwich ELISA for *S. typhimurium.* (**B**) Standard curve of L2-L6 HRP in PBST; (**C**) Specificity of the LPS mAb based sandwich ELISA for *S. typhimurium*; (**D**) Calibration curve of the LPS mAb based sandwich ELISA for *S. typhimurium* in pure milk.

Figure 2A shows that L2-L6HRP is much more sensitive than F1-L6HRP or F1-F1HRP. Notably, we typically found that *S. typhimurium* LPS mAb pairs were more sensitive than any other type of pair. Initial experiments in our study found that when F1 paired with F1HRP, 3–5 ng/mL prepared flagellin could be detected by this sandwich ELISA. By contrast, when L2 paired with L6HRP, only 0.4 ng/mL LPS could be detected (Figure S1, Supporting Information). As the sensitivity of the two sandwich ELISA methods for antigen were comparable, the marked difference in sensitivity for *S. typhimurium* between these two pairs probably results from the properties of the surface of *S. typhimurium*, for which the quantity of LPS is far exceeded by that of flagellin. As reported, *S. typhimurium* usually has 6–8 peritrichous flagella [24]. By contrast, as a major component of the cell wall, a very large number of LPS molecules are present on the surface of *S. typhimurium*.

### 3.4. The Establishment and Cross-Reactivity of the LPS mAb-Based Sandwich ELISA

After optimization, the concentration of coating antibody, detection antibody and Tween 20 in dilution buffer was 5 μg/mL, 2 μg/mL and 0.1% (v/v), respectively. A standard curve for L2-L6HRP was established for *S. typhimurium* after optimization. Figure 2B shows a logistic nonlinear standard curve; under optimal conditions, the detection limit of this sandwich ELISA for *S. typhimurium* is 10^4^ cfu/mL. Previous works of sandwich ELISA with flagellin and LPS MAbs have sensitivity of 10^4^–10^5^ cfu/mL and 10^6^ cfu/mL, respectively [16,25]. Therefore, our sandwich ELISA is more sensitive than the previous works. Figure 2C shows that this LPS mAb-based sandwich ELISA cross-reacts with *S. paratyphi* B, but not with *S. paratyphi* A or *S. enteritidis*. This might indicate that Lps MAbs recognize the O-specific chain of *S. typhimurium* LPS, which is shared by *S. paratyphi* B.

### 3.5. LPS MAbs-Based Immunochromatographic Strip for S. typhimurium

The amount of detection antibody used to conjugate gold nanoparticles, amount of capture antibody on test line and reaction time will significantly affect the performance of the sandwich immunochromatographic strip. For conjugating mAb to gold nanoparticles, we compared 2.5 μg/mL, 5 μg/mL, 10 μg/mL, 15 μg/mL L6 mAb in 1ml colloidal gold (pH 8.0) and found that the color on test line for positive sample increased until the mAb concentration increased to 10 μg/mL, while the test line of negative control was completely blank (data not shown). This indicates the amount of antibody was saturated at the concentration of 10 μg/mL. Therefore, the optimal concentration used for conjugating gold nanoparticles was 10 μg/mL in this study. Similarly, we use 4 mg/mL of L2 mAb to spray the test line of the NC membrane after optimization. For reaction time, we compared 5 min, 10 min and 15 min and found that the sensitivity was similar between 10 min and 15 min, but higher than 5 min. Therefore, the reaction time of this immunochromatographic strip was set at 10 min.

**Figure 3:**
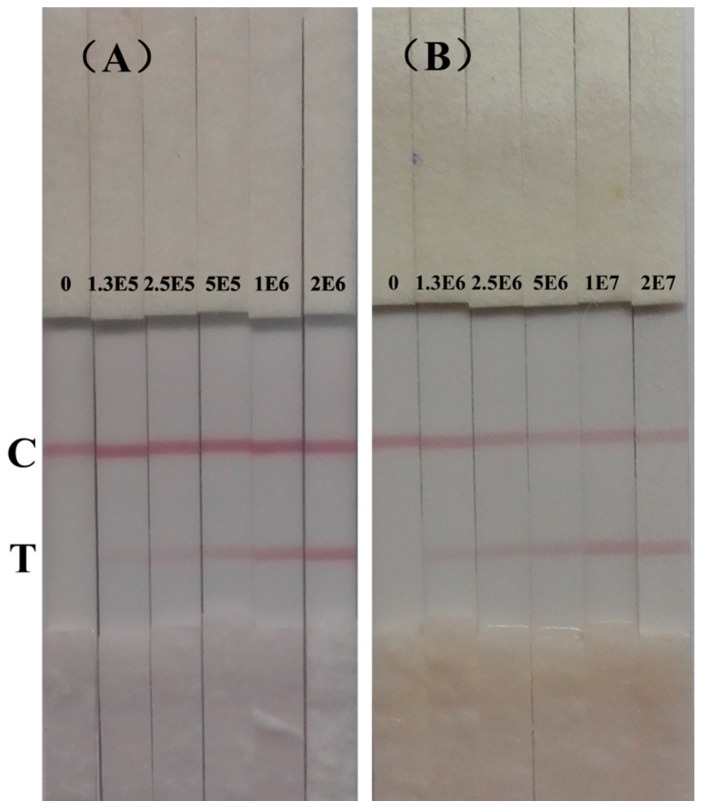
Immunochromatographic strip for detection of *S. typhimurium* (cfu/mL)*.* (**A**) in PBST;(**B**) in pure milk.

Figure 3A shows the results of immunochromatographic strip test of *S. typhimurium* in PBST. The color intensity of test line gradually decreased from 2 × 10^6^ cfu/mL to 1.25 × 10^5^ cfu/mL. With naked eye, the color of the test line at 1.25 × 10^5^ cfu/mL was still red compared with the negative control. Therefore, the detection limit in PBST of this immunochromatographic strip in PBST was 1.25 × 10^5^ cfu/mL. Cross-reactivity of this strip was the same with the developed sandwich ELISA in this study (Figure S2, Supporting Information).

Previous work reported a similar strip for the rapid detection of Salmonella typhi [26]. The strip could detect 1.14 × 10^5^ cfu/mL Salmonella typhi in 15 min with naked eye. Compared with previous work, the developed strip in our study could give the similar sensitivity in just 10 min. What’s more important, the antibody used previous work was polyclonal antibody which prone to be variable from batch to batch when compared with monoclonal antibody. In this study, the rapid immunochromatographic strip is based on highly homogeneous LPS MAbs and will be consistent to detect *S. typhimurium*.

### 3.6. Detection of Milk Samples

In the detection of pure milk samples, we found there is matrix effect in milk samples, even after a 10-fold dilution. However, the absorbance of a similar *S. typhimurium* concentration in pure milk, or in three- or ten-fold diluted milk did not show much difference. Therefore, we choose pure milk as a dilution buffer for generating a calibration curve in sandwich ELISA and directly detect *S. typhimurium* in pure milk. In immunochromatographic strip test, we directly detect *S. typhimurium* in pure milk too and the detection was finished in only 10 min and very stable. What’s more important, the strip can be stored at room temperature for several months and can be low cost point of care device.

Figure 2D shows the calibration curve of the ELISA in pure milk. The detection limit in pure milk was 1.44 × 10^5^ cfu/mL. The mean absorbance of blank pure milk ranges from 0.06 to 0.096. Figure 3B shows the results of immunochromatographic strip test of *S. typhimurium* in spiked pure milk. The test line of negative milk sample was completely blank. The color of test line gradually decreased with the decreasing concentration of *S. typhimurium* and still distinguishable from the negative control until the concentration was at 1.25 × 10^6^ cfu/mL. This indicates the detection limit of this immunochromatographic strip in pure milk was 1.25 × 10^6^ cfu/mL.

Table 3 shows the plate counting method, although very sensitive, involves complex procedures, such as different selective agars, followed by subculture and confirmation by a series of biochemical and serological tests as a result of nonselectivity of this method, which are labor-intensive and time-consuming. However, the developed methods especially the immunochromatographic strip developed in our study was very specific, portable, simple to operate and fast. Spiked pure milk sample with concentration above 1.25 × 10^6^ cfu/mL can be visualized with naked eye in just 10 min without any sophisticated instruments and professional training, which is suitable for high-throughout screening of samples in the food industry. Moreover, the immunosensors intergrated with magnetic separation and nanostructured materials have greatly improved the detection of salmonella in recently years [27]. Our future work will focus on improving the sensitvity of the strip and detecting real sample with low level of *S. typhimurium* after enrichment.

**Table 3:** Comparison between sandwich ELISA, immunochromatographic strip and plate counting method for detection of spiked milk samples.

	Sandwich ELISA	Immunochromatographic	Plate Counting
Sensitivity	(1.83 ± 0.32) × 10^5^	1.25 × 10^6^	≤(1.9 ± 0.32) × 10^4^
Detection time	3 h	10 min	24 h
Specificity	Specific	Specific	-
Portability	-	Portable	-
Simplicity of operation	-	Very simple	-

## 4. Conclusions

In this study, H:i flagellin from *S. typhimurium* was purified and murine flagellin MAbs were selected. LPS MAbs were produced with smooth LPS from *S. typhimurium* mixed with boiled *S. typhimurium* that was used as an immunogen. We characterized four flagellin MAbs, named F1, F2, F3 and F4, and six LPS MAbs, named L1, L2, L3, L4, L5 and L6. Then, both flagellin and LPS MAbs were paired in a sandwich ELISA format and were further compared for the detection of *S. typhimurium*. We found that LPS mAb pairs tended to be much more sensitive than any other combination of pairs. A sandwich ELISA and immunochromatographic strip based on LPS MAbs were established and assessed for the sensitive detection of *S. typhimurium*. The sandwich ELISA can detect 10^4^ cfu/mL *S. typhimurium* in PBST and 1.44 × 10^5^ cfu/mL in artificially spiked milk samples. The immunochromatographic strip can detect 1.25 × 10^5^ cfu/mL *S. typhimurium* in PBST and 1.25 × 10^6^ cfu/mL in artificially spiked milk samples. The developed LPS mAb-based sandwich ELISA and immunochromatographic strip could be effective methods for the sensitive detection of *S. typhimurium* in food samples.

## Supplementary Files

Supplementary File 1
