# Supplementary material for: A Highly Sensitive ELISA and Immunochromatographic Strip for the Detection of Salmonella typhimurium in Milk Samples
Source: Sensors (Basel). 2015 Mar 4;15(3):5281–92. doi: 10.3390/s150305281 (PMC4435159; doi:10.3390/s150305281)
Supplement: Supplementary File 1 [file sensors-15-05281-s001.pdf]

OPEN ACCESS

sensors

ISSN 1424-8220
www.mdpi.com/journal/sensors

Supplementary Information

A Highly Sensitive ELISA and Immunochromatographic Strip for the Detection of *Salmonella typhimurium* in Milk Samples. *Sensors* 2015, *15*, 5281-5292

Wenbin Wang, Liqiang Liu, Shanshan Song, Lijuan Tang, Hua Kuang and Chuanlai Xu *

State Key Lab of Food Science and Technology, School of Food Science and Technology, Jiangnan University, Wuxi 214122, China; E-Mails: wenbin66@yeah.net (W.W.); raxray@gmail.com (L.L.); songshanshan0626@126.com (S.S.); tlj844944327@163.com (L.T.); kuangh@jiangnan.edu.cn (H.K.)

* Author to whom correspondence should be addressed; E-Mail: xcl@jiangnan.edu.cn; Tel: +86-510-8532-9076.

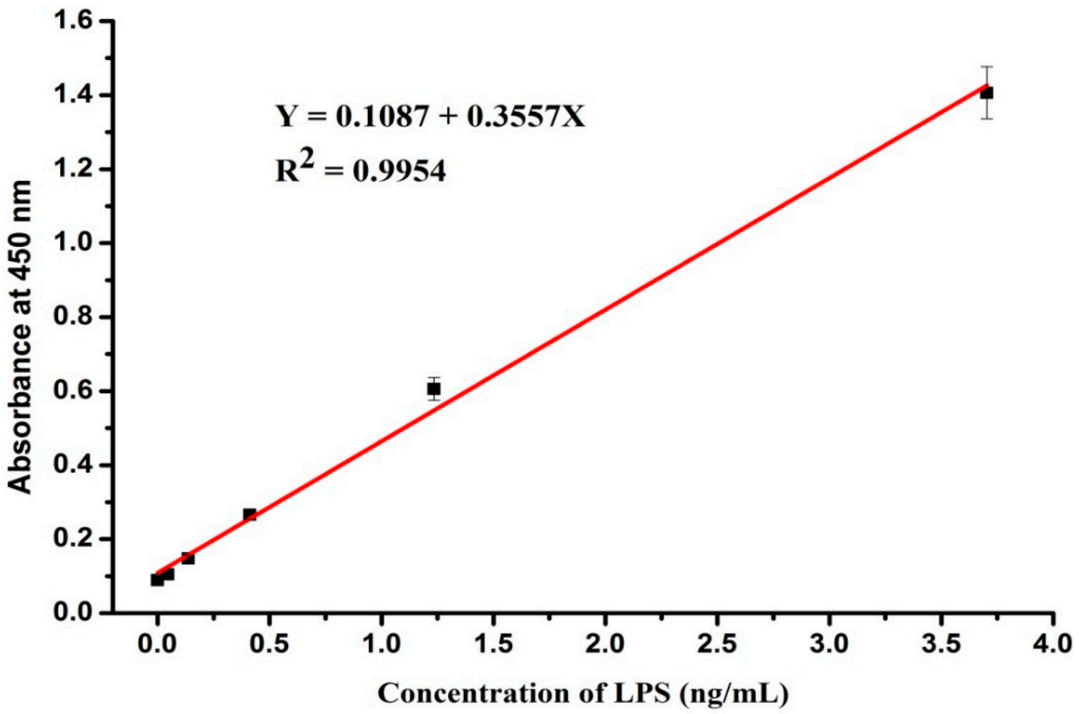

Figure S1. Standard curve of L2-L6 HRP for LPS in PBST.

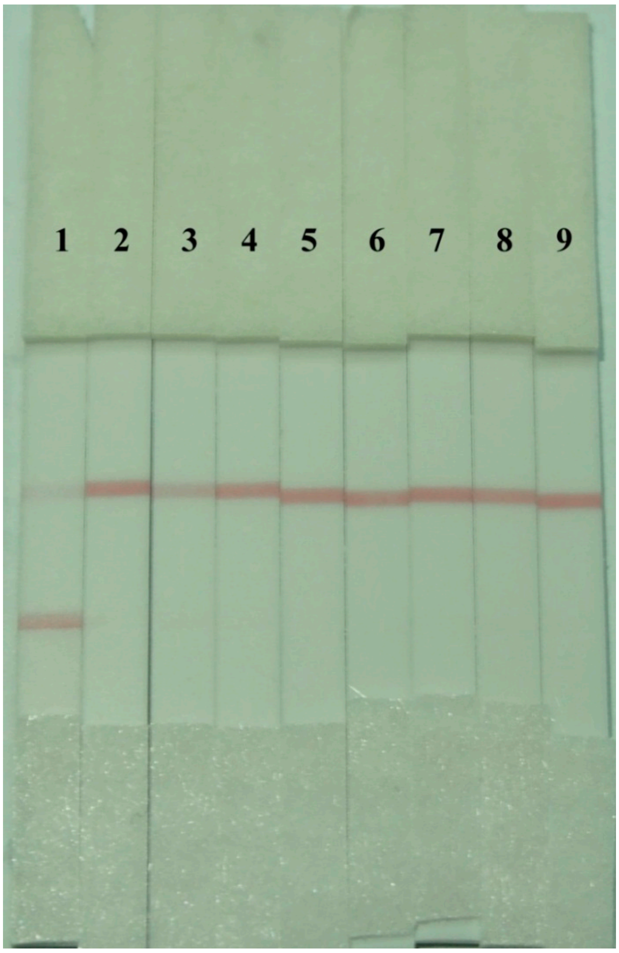

Figure S2. Specificity of the immunochromatographic strip for *S. typhimurium*. 1 to 9 was correspond to paratyphi B, *S. paratyphi* A, *S. enteritidis*, *Escherichia coli* O157, *Staphylococcus aureus*, *Listeria monocytogenes*, and *Cronobacter sakazakii*, *Campylobacter jejuni* and PBS. Concentrations were all at 10^8 cfu/mL.

© 2015 by the authors; licensee MDPI, Basel, Switzerland. This article is an open access article distributed under the terms and conditions of the Creative Commons Attribution license (http://creativecommons.org/licenses/by/4.0/).
